# Mitochondrial (Dys)function and Insulin Resistance: From Pathophysiological Molecular Mechanisms to the Impact of Diet

**DOI:** 10.3389/fphys.2019.00532

**Published:** 2019-05-03

**Authors:** Domenico Sergi, Nenad Naumovski, Leonie Kaye Heilbronn, Mahinda Abeywardena, Nathan O’Callaghan, Lillà Lionetti, Natalie Luscombe-Marsh

**Affiliations:** ^1^ Nutrition and Health Substantiation Group, Nutrition and Health Program, Health and Biosecurity, Commonwealth Scientific and Industrial Research Organisation (CSIRO), Adelaide, SA, Australia; ^2^ Adelaide Medical School, The University of Adelaide, Adelaide, SA, Australia; ^3^ Faculty of Health, University of Canberra, Canberra, ACT, Australia; ^4^ Collaborative Research in Bioactives and Biomarkers (CRIBB) Group, Canberra, ACT, Australia; ^5^ Department of Chemistry and Biology “A. Zambelli”, University of Salerno, Fisciano, Italy

**Keywords:** mitochondrial function, lipotoxicity, oxidative metabolism, insulin resistance, skeletal muscle

## Abstract

Mitochondrial dysfunction has been implicated in the pathogenesis of insulin resistance, the hallmark of type 2 diabetes mellitus (T2DM). However, the cause-effect relationship remains to be fully elucidated. Compelling evidence suggests that boosting mitochondrial function may represent a valuable therapeutic tool to improve insulin sensitivity. Mitochondria are highly dynamic organelles, which adapt to short- and long-term metabolic perturbations by undergoing fusion and fission cycles, spatial rearrangement of the electron transport chain complexes into supercomplexes and biogenesis governed by peroxisome proliferator-activated receptor γ co-activator 1α (PGC 1α). However, these processes appear to be dysregulated in type 2 diabetic individuals. Herein, we describe the mechanistic link between mitochondrial dysfunction and insulin resistance in skeletal muscle alongside the intracellular pathways orchestrating mitochondrial bioenergetics. We then review current evidence on nutritional tools, including fatty acids, amino acids, caloric restriction and food bioactive derivatives, which may enhance insulin sensitivity by therapeutically targeting mitochondrial function and biogenesis.

## Introduction

Obesity has reached epidemic proportions worldwide and its incidence is on the rise affecting both adults and children^1^. Obesity is strongly associated with type 2 diabetes mellitus (T2DM), non-alcoholic fatty liver disease, certain types of cancers and poorer mental health (Hotamisligil, 2006; Brown et al., 2009; Luchsinger and Gustafson, 2009). The association between obesity and metabolic dysfunctions is predominantly dictated by fat distribution with increased visceral or intra-abdominal fat being more detrimental to metabolic health compared to peripheral adiposity depots, which appear to confer a better metabolic profile (Vazquez et al., 2007; Hayashi et al., 2008; Castro et al., 2014). Of note, obesity and visceral fat accumulation in particular are underlain by a low-grade chronic inflammation (Hotamisligil, 2006) and increased ectopic fat storage in metabolically active tissues including skeletal muscle and liver, a phenomenon termed lipotoxicity (Unger, 2002). Remarkably, these pathophysiological features of obesity represent the main mechanisms bridging the gap between increased fatty acid availability, sustained by enhanced adipose tissue lipolysis and impaired fatty acid beta-oxidation, and insulin resistance, the hallmark of T2DM. Mitochondrial dysfunction and the subsequent impairment in metabolic fuel oxidation appear to be the metabolic culprit underlying the accumulation of lipotoxic lipid metabolites. In support of this notion, a decrease in fatty acid oxidation induces the buildup of ceramide and diacylglycerol, which have been shown to impair the insulin signal transduction pathway (Samuel et al., 2010).

Herein, we describe the relationship among mitochondrial dysfunction, lipotoxicity and insulin resistance in skeletal muscle. Particularly, we focus on the regulation of mitochondrial function, intended as the ability of mitochondria to adapt to the availability and oxidase metabolic fuels, mitochondrial biogenesis and post-translational modifications of the proteins orchestrating mitochondria bioenergetics. Furthermore, we will describe dietary factors known to be detrimental to mitochondrial health and nutrients as well as bioactive food derivatives, which, instead, appear to be able to boost mitochondrial function.

## Insulin Signalling Pathway

**Figure 1:**
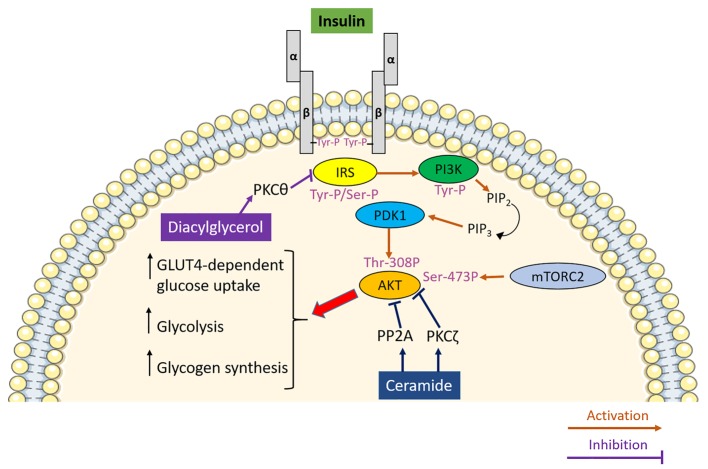
The role of lipotoxicity in promoting insulin resistance in skeletal muscle. Insulin upon binding to its receptor activates a signal transduction pathway culminating in the PDK1 and mTORC2-mediated phosphorylation and activation of AKT, which, by modulating its downstream effectors, promotes glucose uptake, glycolysis and glycogen synthesis in skeletal muscle. Diacylglycerol inhibits insulin signalling by activating protein kinase C θ (PKCθ) which phosphorylates insulin receptor substrate (IRS) on serine residues, thereby inhibiting it. Ceramide impedes insulin signalling *via* two separate mechanisms involving PKCζ-induced phosphorylation and protein phosphatase 2A (PP2A)-mediated dephosphorylating of AKT.

Insulin is a peptide hormone secreted by the pancreatic β cells located in the islet of Langerhans. Insulin exerts its role in the regulation of whole body metabolism by binding to a cell surface receptor, which belongs to a subfamily of receptor tyrosine kinases and is characterised by two extracellular ligand-binding α subunits and two intracellular tyrosine-kinase β subunits (Lee et al., 2014). Upon binding to its cognate receptor, insulin induces conformational changes by bringing the α subunits closer and inducing the autophosphorylation of tyrosine residues mediated by the β subunits (Ryder et al., 2001). The β subunits of the insulin receptor are responsible for the phosphorylation of insulin receptor substrate (IRS) at tyrosine residues (Sun and Rothenberg, 1991), which in turn promote the interaction between IRS and proteins containing SRC homology 2 (SH2) domains (Koch et al., 1991) including phosphoinositide 3-kinase (PI3K; Myers et al., 1992). PI3K is a heterodimeric protein, which consists of two subunits, the p85 regulatory subunit, which contains the SH2 domain and is involved in IRS-PI3K interaction (Skolnik et al., 1991), and the p110, which contains the catalytic subunit of the enzyme (Hiles et al., 1992). PI3K catalyses the phosphorylation of phosphatidylinositol 4,5-bisphosphate [PI(4,5)P2] and its conversion to phosphatidylinositol 3,4,5-trisphosphate [PI(3,4,5)P3], which in turn, by activating 3-phosphoinositide-dependent protein kinase (PDK) 1, ultimately leads to the activation of AKT (Walker et al., 1998). The activation of AKT requires a dual serine/threonine phosphorylation mediated by PDK1 (threonine 308) and mammalian target of rapamycin complex 2 (mTORC2) (serine 473), respectively (Figure 1; Kumar et al., 2008). Once activated, AKT phosphorylates downstream targets including forkhead box O1 (FOXO1), glycogen synthase kinase 3 (GSK3), AKT substrate of 160 kDa (AS160) and mammalian target of rapamycin (mTOR; Taniguchi et al., 2006), which are pivotal in mediating the metabolic effects of insulin (Figure 1). Although the regulation of metabolic fuel homeostasis results from the integration of insulin signalling at different organs and tissues encompassing liver (Petersen et al., 1998; Leavens and Birnbaum, 2011; Perry et al., 2015; Samuel and Shulman, 2016), skeletal muscle, adipose tissue (Anthonsen et al., 1998; Virtanen et al., 2002) and the brain (Tups et al., 2017), the main focus of this review will be on skeletal muscle.

Skeletal muscle is pivotal in glucose homeostasis and energy metabolism in light of its capacity to take up and metabolise approximately 80% of postprandial circulating glucose (Shulman et al., 1990). The rate-limiting step in insulin-mediated glucose uptake and consequent intracellular metabolic processing by the skeletal muscle is the translocation of the glucose transporter type 4 (GLUT-4) at the cell surface. As described earlier, insulin upon binding to its cognate receptor initiates a phosphorylation cascade, which culminates with the phosphorylation and activation of AKT, which in turn phosphorylates AS160 promoting GLUT4-containing storage vesicles (GSVs) trafficking to the cell membrane (Bruss et al., 2005). Muscle glycogen synthesis also involves AKT-induced phosphorylation and inhibition of GSK3 resulting in increased glycogen synthase activity (Figure 1; Jensen and Lai, 2009). Nonetheless, despite the importance of GLUT-4 in insulin-induced glucose uptake in skeletal muscle, glucose can enter the myocytes with mechanisms independent of insulin, which rely upon the activation of the energy sensor 5′ adenosine monophosphate-activated protein kinase (AMPK; O’Neill et al., 2011; Friedrichsen et al., 2013). Indeed, mice with targeted deletion of the insulin receptor in skeletal muscle preserve muscle contraction-induced glucose uptake (Wojtaszewski et al., 1999) despite displaying impaired insulin-mediate glucose uptake in skeletal muscle (Kim et al., 2000b). Considering the central role of skeletal muscle in the control of glucose homeostasis and the fact that insulin resistance in skeletal muscle is evident decades before β-cell failure and overt hyperglycaemia (Lillioja et al., 1988; Warram et al., 1990), skeletal muscle represents an ideal target for the treatment of T2DM.

## Lipotoxicity and Insulin Resistance

Insulin resistance is the hallmark of T2DM aetiology. It is referred to as a blunted response of metabolically active tissues to insulin leading to a dysregulation of nutrient fluxes, metabolism and homeostasis. At the molecular level, the ectopic accumulation of lipids and lipid secondary metabolites in metabolically active tissues, and particularly skeletal muscle, represents a major determinant of insulin resistance. In support of this notion, intramyocellular lipids represent a better predictor of muscle insulin resistance compared to adiposity in young, sedentary, lean subjects (Krssak et al., 1999). However, the accumulation of intramyocellular lipids itself is not sufficient to explain the association between ectopic lipid accumulation and insulin resistance. Indeed, athletes are highly insulin-sensitive in spite of increased intramyocellular lipid mainly stored in the form of triglycerides (Goodpaster et al., 2001), which led to the formulation of the so-called athlete paradox. The athlete paradox provides insights into the relationship between intramyocellular lipid and insulin resistance, highlighting that the detrimental effect of lipids on insulin sensitivity is dependent on the accumulation of reactive lipid species such as diacylglycerols and ceramides rather than accumulation of lipids in the form of triglycerides *per se* (Dresner et al., 1999; Yu et al., 2002; Samuel and Shulman, 2012; Kitessa and Abeywardena, 2016). Diacylglycerols are lipid intermediates that signal *via* protein kinase C (PKC). Particularly, the lipotoxic buildup of diacylglycerol in skeletal muscle results in sustained activation of PKCθ (Yu et al., 2002), which in turn phosphorylates IRS on serine residues hampering insulin-mediated tyrosine-phosphorylation and therefore promoting insulin resistance (Figure 1; Li et al., 2004). Importantly, this mechanism has also been confirmed in humans supporting the pathophysiological relevance of diacylglycerol-induced insulin resistance beyond rodent models (Itani et al., 2002). As well as diacylglycerol, ceramide also contributes to insulin resistance. The deleterious effect of ceramide on insulin signalling results from its ability to block the activation of AKT *via* two independent mechanisms (Chavez and Summers, 2012). The first mechanism involves the phosphorylation of AKT on pleckstrin homology domain by PKCζ, which in turn is activated by ceramide. This lowers the affinity of AKT for phosphoinositide (Powell et al., 2003) and blocks AKT translocation to the plasma membrane (Stratford et al., 2001). By contrast, dephosphorylation of AKT by protein phosphatase 2A (PP2A) underlies the second mechanism linking intracellular ceramide accumulation to insulin resistance (Figure 1; Chavez et al., 2003).

## Mitochondrial Dysfunction and Insulin Resistance

**Figure 2:**
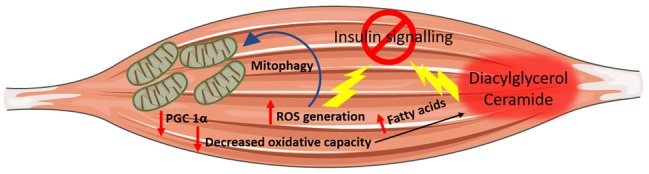
Mitochondrial dysfunction and insulin resistance. Impaired mitochondria oxidative capacity leads to a decrease in metabolic substrate catabolism resulting in increased intramyocellular fatty acids availability, which may be channelled towards lipotoxic lipid species biosynthesis (i.e., ceramide and diacylglycerol) both of which have been associated with insulin resistance. Increased nutrient supplies also induce an increase in mitochondrial reactive oxygen species (ROS) production, which can directly induce insulin resistance and elicit oxidative damage to mitochondrial DNA, protein and lipid promoting the removal of damaged mitochondria by mitophagy.

A decrease in metabolic substrate oxidation appears as a primary defect, which, by triggering a cascade of events culminating with the intracellular accumulation of the diacylglycerol and ceramide, hampers insulin signalling and promotes insulin resistance. Mitochondria, in light of their pivotal role in oxidative metabolism, have been identified as the cellular organelles at the interphase between impaired fuels, and particularly fatty acids oxidation, lipotoxicity and insulin resistance. This intuitive association between impaired mitochondrial oxidative capacity and insulin resistance has been confirmed in landmark studies, which described an impairment in mitochondrial function in individuals diagnosed with T2DM. The preliminary evidence linking mitochondrial dysfunction to insulin resistance comes from studies performed in obese and insulin-resistant individuals who exhibited a decrease in skeletal muscle mitochondria oxidative capacity and defective lipid metabolism compared to healthy, lean controls (Kelley et al., 1999; Simoneau et al., 1999; Kim et al., 2000a). Furthermore, individuals with T2DM have reduced NADH_2_-O_2_ oxidoreductase activity (Kelley et al., 2002), which further supports the association between T2DM and mitochondrial dysfunction proposing the latter as an underlying defect in the pathogenesis of insulin resistance. Microarray studies have successively strengthened this association by providing evidence that genes involved in oxidative metabolism and under the control of PGC 1α are downregulated in the skeletal muscle of individuals with a family history of T2DM and patients affected by T2DM compared to healthy controls (Mootha et al., 2003; Patti et al., 2003). Furthermore, assessment of mitochondria function *in vivo* using non-invasive measurement of the phosphocreatine resynthesis rate after exercise corroborated these remarkable mitochondrial defects at the protein as well as at the transcriptional levels providing further evidence that muscle mitochondria oxidative capacity is impaired in patients with overt T2DM (Schrauwen-Hinderling et al., 2007). Most importantly, a decrease in mitochondrial respiration was reported in the first-degree relatives of individual with T2DM (Phielix et al., 2008) indicating that mitochondrial dysfunction may precede the onset of full blown T2DM. Mitochondrial dysfunction appears to be a direct consequence of intrinsic mitochondrial defect at the level of the oxidative phosphorylation system and the electron transport chain rather than a decrease in mitochondrial content assessed by mitochondria DNA copy number. In support of this, a 35% decrease in ADP-stimulated mitochondrial respiration was reported in patients with T2DM after normalisation for mitochondrial content (Phielix et al., 2008). Thus, the decrease in mitochondrial oxidative capacity and the consequent impairment in metabolic fuel oxidation provide a plausible cause-effect association linking mitochondrial dysfunction to the accumulation of lipotoxic lipid intermediates, which culminates with the development of insulin resistance (Figure 2). Despite this paradigm being attractive, a potential caveat applies to this model. Indeed, skeletal muscle is characterised by an enormous spare respiratory capacity allowing for a 10–20-fold increase in oxygen consumption above resting, which denies the possibility that a decrease in mitochondrial function of the magnitude observed in type 2 diabetic individuals might lead to the accumulation of diacylglycerol and ceramide (Holloszy, 2008). Nonetheless, skeletal muscle can call upon its spare respiratory capacity in situations of increased energy demand (i.e., exercise), which triggers intracellular signalling pathways aimed at increasing mitochondria biogenesis and efficiency to match increased ATP demand. Thus, in the absence of stimuli that trigger mitochondrial adaptation to match increased energy demand, as in response to AMPK activation, even slight changes in mitochondria oxidative capacity, if protracted over time, will result in ectopic lipid accumulation (Figure 2; Montgomery and Turner, 2015).

Another putative mechanism linking mitochondrial dysfunction to insulin resistance is represented by the generation of reactive oxygen species (ROSs) by mitochondria. ROSs are mandatory by-products of mitochondrial energy metabolism with their production being counterbalanced by the intracellular antioxidant system. However, when ROS production overwhelms cellular antioxidant capacity, oxidative stress occurs (Schieber and Chandel, 2014). An increase in electron donors derived from nutrient oversupply and catabolism increases electron supply to the mitochondrial electron transport chain, which in turn induces a high proton gradient across the inner mitochondrial membrane, which, if not coupled to an increase in ATP synthesis, culminates in greater ROS production. ROSs, apart from inducing oxidative damage to nuclear DNA, lipids and protein (Cross et al., 1987), are also signalling molecules that can directly induce insulin resistance (Figure 2; Anderson et al., 2009). The increase in ROS generation and the parallel oxidative stress are not without consequence for mitochondria oxidative metabolism. Indeed, ROS can directly induce oxidative damage to mitochondrial DNA, protein and lipids and consequently trigger the removal of damaged mitochondria by mitophagy (Figure 2). Thus, oxidative stress not only interferes with the insulin signal transduction pathway and promotes insulin resistance directly (Anderson et al., 2009) but can also hamper insulin signalling indirectly by inducing mitochondrial damage and mitophagy. The consequent decrease in mitochondrial function and density compromises overall cellular oxidative capacity, thereby favouring the ectopic accumulation of lipotoxic lipid intermediates.

Despite the well-documented association between mitochondrial dysfunction and insulin resistance, whether impaired mitochondrial oxidative capacity is causal to or is a consequence of insulin resistance remains a matter of debate with several authors failing to confirm this association. Particularly, mitochondrial function has not been reported to be compromised in obese or type 2 diabetic individuals (Trenell et al., 2008). Furthermore, while overfeeding sedentary non-obese individuals for 28 days induced insulin resistance, it did not affect the protein level of PGC 1α, complex I, II and V of the electron transport chain supporting the possibility that insulin resistance arises independently from mitochondrial dysfunction (Samocha-Bonet et al., 2012). Severe hyperglycaemia has been shown to reversibly decrease mitochondria respiration in human cultured myotubes (Rabøl et al., 2009), which supports the possibility that mitochondrial dysfunction may arise in response to sustained hyperglycaemia and energetic substrates oversupply to skeletal muscle. The detrimental effect of hyperglycaemic glucotoxicity on mitochondria has also been recently described in pancreatic β cells with glucotoxicity decreasing mitochondrial oxygen consumption rate by inducing the upregulation of voltage-dependent anion channel-1 (Zhang et al., 2019). However, whether this mechanism holds true in skeletal muscle remains to be elucidated. Animal studies aimed at shedding the light on the role of mitochondrial function in the pathogenesis of insulin resistance also failed to confirm a direct cause-effect relationship between insulin resistance and defective mitochondrial oxidative metabolism. A high-fat diet has been widely described to induce insulin resistance in rodent models (Williams et al., 2014), and it has also been used to elucidate the relationship between the metabolic insult elicited by increased fatty acids supply, insulin resistance and mitochondrial function. Similar to humans, diet-induced insulin resistance in rats and mice was not associated with a decrease in skeletal muscle mitochondrial proteins. Instead, the high-fat diet induced a gradual increase in skeletal muscle mitochondria as indicated by an increase in mitochondrial DNA (mtDNA) and proteins with a mechanism dependent on peroxisome proliferator-activated receptor δ (PPARδ) activation and upregulation of PGC 1α (Hancock et al., 2008). Furthermore, animals fed a high-fat diet displayed increased fatty acid oxidative capacity (Turner et al., 2007) and elevation of mitochondrial proteins involved in oxidative metabolism (Garcia-Roves et al., 2007). According to the evidence described so far, the relationship between insulin resistance and mitochondrial dysfunction can be exemplified by three different scenarios: impaired mitochondrial function is associated with insulin resistance, insulin resistance develops despite mitochondrial function remaining unaffected and finally mitochondria oxidative capacity is increased despite the development of diet-induced insulin resistance. Thus, mitochondrial dysfunction does not appear to be a prerequisite for the onset of insulin resistance. A possible explanation to this conundrum is the compensatory increase in mitochondrial function to offset fatty acid oversupply and prevent their deleterious effects of insulin signalling. While this protective mechanism may prevent researchers from detecting slight, but significant defects in mitochondria biology, the time course and the magnitude of this adaptation may not suffice to counteract the increase in fatty acid availability and prevent ectopic lipid accumulation (Montgomery and Turner, 2015). In agreement with this, upregulating PGC 1β or activating heat shock protein 72 to increase mitochondrial function above the physiological adaptive response triggered by lipid oversupply mitigate lipid-induced insulin resistance (Wright et al., 2011; Henstridge et al., 2014). Furthermore, lipid infusion induced a 30% decrease in insulin sensitivity in endurance athletes compared to a 70% decrease in sedentary controls suggesting that higher mitochondrial oxidative capacity, typical of endurance athletes, partially protects against lipid-induced insulin resistance (Phielix et al., 2012). Thus, independently of whether mitochondrial dysfunction is a cause or a consequence of insulin resistance, boosting mitochondrial function remains a promising strategy to improve insulin sensitivity.

## Regulation of Mitochondrial Biogenesis

**Figure 3:**
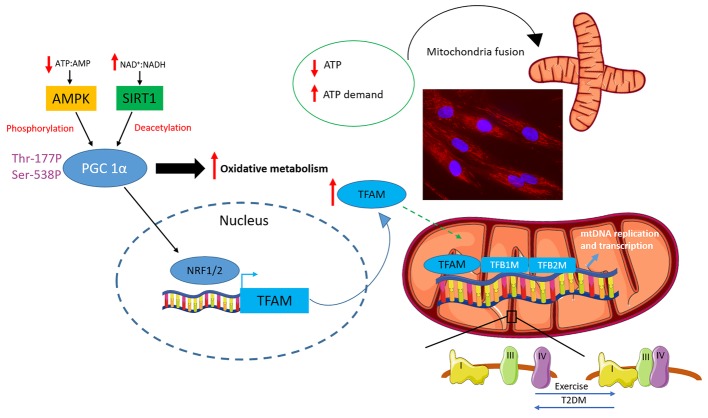
Regulation of mitochondrial biogenesis, fusion and supercomplex formation. An increase in energy demand/decrease energy availability marked by a decrease in ATP:AMP and an increase in NAD+:NADH ratios is sensed by 5′ adenosine monophosphate-activated protein kinase (AMPK) and sirtuin-1 (SIRT1), respectively. These energy gauges activate the master regulator of mitochondrial oxidative metabolism and biogenesis: peroxisome proliferator-activated receptor γ co-activator 1α (PGC 1α). PGC 1α activates the nuclear respiratory factors 1 and 2 (NRF-1 and NRF-2), which in turn promotes the transcription of mitochondrial transcription factor A (TFAM). TFAM directly interacts with mitochondrial DNA (mtDNA) and, in concert with mitochondrial transcription specificity factors TFB1M and TFB2M, regulates mtDNA replication and transcription. Mitochondrial function is also modulated by shifts in mitochondria dynamics with a drop in ATP levels or an increase in ATP demand triggering mitochondria fusion also depicted in the fluorescent microscopy picture of myotube mitochondria stained with Mitotracker red (Thermo Fisher Scientific) (cells were purchased from Cook Myosite, USA). Finally, mitochondrial complexes can assemble into multimeric super assembled structures termed supercomplexes. The most abundant mitochondrial supercomplex is made up of complex I, III and IV also termed respirasome. Exercise has been shown to promote supercomplex formation, which, instead, was reported to be decreased in individuals affected by type 2 diabetes (T2DM).

A key peculiarity of mitochondria is that they have their own circular DNA, referred to as mtDNA (Schatz et al., 1964), which encodes 22 transfer RNAs and 13 proteins required for mitochondrial respiration. Nonetheless, the majority of genes involved in mitochondrial metabolism and biogenesis are nuclear-encoded genes whose transcription, translation and transport into the mitochondria occur in concomitance and are coordinated with mtDNA replication, transcription and translation. Key in orchestrating this process is the nuclear-encoded mitochondrial transcription factor A (TFAM), which is pivotal in regulating mtDNA transcription by directly interacting with mitochondrial genome along with mitochondrial transcription specificity factors TFB1M and TFB2M (Figure 3; Gleyzer et al., 2005; Ljubicic et al., 2010). The expression of TFAM is under the control of the nuclear respiratory factors 1 and 2 (NRF-1 and NRF-2), which promote mitochondrial biogenesis by inducing TFAM-dependent mtDNA replication and transcription (Figure 3; Gordon et al., 2001; Gleyzer et al., 2005; Picca and Lezza, 2015). The activation of NRF-1 and 2 and the subsequent induction of TFAM are governed by the master regulator of mitochondrial metabolism and biogenesis: PGC 1α (Figure 3). PGC 1α is a transcription coactivator, which, by interacting with a broad range of transcription factors, regulates the expression of key genes involved in mitochondrial biogenesis, adaptive thermogenesis and metabolic substrate metabolism (Liang and Ward, 2006). Besides its role in mitochondrial biogenesis, PGC 1α plays a pivotal role in promoting fatty acid β-oxidation by functioning as a coactivator for PPARα and δ, which in turn regulates the expression of genes involved in mitochondria fatty acid catabolism (Vega et al., 2000; Wang et al., 2003). Thus, in light of its role in promoting mitochondrial biogenesis and regulating fatty acid β-oxidation, PGC 1α may be at the interphase among mitochondrial dysfunction, ectopic accumulation of lipotoxic lipid metabolites and insulin resistance. Further supporting the key role of this transcription factor, genes involved in oxidative metabolism that are downregulated in individuals with T2DM are under the control of PGC 1α (Patti et al., 2003). PGC 1α overexpression enhances insulin sensitivity in human muscle cells promoting a phenotype resembling that of highly trained athletes. These cells are characterised by an increase in mitochondrial density and are protected against lipotoxicity with excess lipids being stored in inert lipid droplets (Koves et al., 2013). In support of this notion, the insulin sensitising effect of exercise (Meex et al., 2010) is paralleled by a concomitant exercise-induced upregulation of PGC 1α (Uguccioni et al., 2010). Furthermore, the insulin sensitising drug, rosiglitazone, restores PGC 1α expression in type 2 diabetic individuals with the effect of this drug on insulin sensitivity being coupled with enhanced muscular oxidative capacity and restoration of oxidative metabolism transcriptome towards the values typical of metabolic healthy individuals (Mensink et al., 2007). Thus, not only PGC 1α upregulation is paralleled by an improvement in insulin sensitivity, but also PGC 1α-dependent defects in mitochondrial oxidative metabolism are reversible confirming this transcription cofactor as a valuable target to restore insulin sensitivity and the aberrant transcriptional control of mitochondrial biogenesis (Mootha et al., 2003; Patti et al., 2003).

The activity of PGC 1α is regulated by both phosphorylation and deacetylation (Figure 3). PGC 1α phosphorylation and activation are governed by the AMPK (Vaughan et al., 2014), an ancient energy gauge, which in turn is activated to rewire energy metabolisms in response to a drop in ATP:AMP and ATP:ADP ratios (Kahn et al., 2005). AMPK is a heterotrimeric protein encompassing a catalytic subunit α and two regulatory subunits β and γ (Herzig and Shaw, 2018). The α subunit is phosphorylated at Thr172 by upstream kinases including calcium-sensitive kinase CAMKK2 (Fogarty et al., 2010) and liver kinase B1 (Shackelford and Shaw, 2009) leading to the activation of the AMPK complex (Herzig and Shaw, 2018). The regulatory subunit β contains a carbohydrate-binding domain, which allows AMPK to function as a glycogen sensor, while the γ subunit detects changes in energy availability by binding to AMP and, to a lesser extent, to ADP. Particularly, when ATP:AMP and ATP:ADP ratios drop, as in response to exercise, AMPK is activated to restored energy status by inhibiting energy consuming anabolic reactions while promoting catabolic reactions including oxidation of metabolic substrates in mitochondria (Kahn et al., 2005). Indeed, AMPK inhibits gluconeogenesis, *de novo* fatty acid and protein synthesis as well as cell growth. It inhibits fatty acid and sterol synthesis by phosphorylating acetyl-CoA carboxylase, which catalyses the first step in the *de novo* lipid synthesis and hydroxymethylglutaryl-CoA reductase the rate-limiting step in cholesterol synthesis, whereas the phosphorylation of glycogen synthases 1 and 2 underpins AMPK-mediated inhibition of glycogen synthesis (Herzig and Shaw, 2018). Finally, AMPK inhibits both cell growth and protein synthesis, two major consumers of ATP, by inhibiting mTOR complex 1 (Gwinn et al., 2008). While inhibiting anabolic pathways, AMPK activates catabolic pathways aimed at restoring cellular ATP levels. AMPK promotes glucose utilisation by stimulating its uptake and metabolism *via* glycolysis, and it also induces fatty acid β-oxidation by phosphorylating and inhibiting acetyl-CoA carboxylase, thereby leading to a decrease in malonyl-CoA and the consequent activation of carnitine palmitoyl acyl transferase 1, which results in increased fatty acid import into the mitochondria where they are catabolised (Herzig and Shaw, 2018). The effect of AMPK on metabolic substrate catabolism and its ability to induce a cellular metabolic reprogramming in response to a decrease in energy availability/increase energy demand also relies on the capacity of AMPK to promote mitochondria biogenesis and regulate mitochondria dynamics and mitophagy (Herzig and Shaw, 2018). Furthermore, besides its role in promoting PGC 1α phosphorylation AMPK also increases its expression (Garcia-Roves et al., 2008; Vaughan et al., 2014). AMPK also promotes, albeit indirectly, PGC 1α deacetylation and activation, which is mediated by the NAD-dependent protein deacetylase sirtuin-1 (SIRT1) whose activation is a direct consequence of the increase in NAD^+^:NADH ratio (Canto and Auwerx, 2009). Particularly, AMPK has been shown to promote SIRT1-dependent PGC 1α deacetylation by increasing intracellular NAD^+^ levels and leading to the subsequent activation of SIRT1 with this mechanism being proposed as key in allowing the cell to switch between different metabolic substrates (Cantó et al., 2010).

## Protein Acetylation as a Regulator of Mitochondrial Function

The regulation of mitochondria biogenesis and the expression of genes orchestrating oxidative metabolism are not the only determinant of mitochondrial function. Indeed, mitochondrial activity and oxidative capacity are also regulated by post-transcriptional modifications of the mitochondria electron transport chain complexes and mitochondrial enzymes involved in oxidative metabolism. Acetylation of mitochondrial proteins has been described as a pivotal post-transcriptional modification in governing mitochondrial metabolism as shown by the acetylation of the intermediate metabolic enzymes involved in the tricarboxylic acid cycle and fatty acid β-oxidation (Zhao et al., 2010). Additionally, acetylation is regulated by perturbation of energy metabolism as demonstrated by an increase in mitochondrial protein acetylation upon fasting (Kim et al., 2006) and caloric restriction (Schwer et al., 2009) in mice. Counterintuitively, a high-fat diet also increases mitochondrial protein acetylation (Hirschey et al., 2011). Thus, mitochondrial protein acetylation is susceptible to changes in energy or nutrient availability independently on whether such metabolic perturbation arises from nutrient deficiency or excess. However, despite an increase in protein acetylation in both the fed and fasted state, these metabolic states promote different acetylation patterns. While 62% of mitochondrial proteins were found to be acetylated in fed and fasted animals, the 14% of acetylated proteins were unique to fed mice, whereas 24% were unique to fasted animals (Kim et al., 2006). Particularly, hyperacetylated mitochondrial proteins have reduced activity resulting in mitochondrial dysfunction and lower ATP production (Anderson and Hirschey, 2012). Mitochondrial hyperacetylation, and the downstream decrease in mitochondrial function, also negatively impacts on fatty acid β-oxidation (Anderson and Hirschey, 2012) suggesting mitochondrial protein hyperacetylation as a putative mechanism underpinning ectopic lipid accumulation and insulin resistance. A pivotal regulator of mitochondrial protein acetylation, and thereby energy metabolism, is the mitochondrial NAD-dependent protein deacetylase SIRT3. SIRT3 plays a central role in the regulation of mitochondrial and energy metabolism with its deletion in mice exacerbating the deleterious effect of a high-fat diet on metabolic health resulting in accelerated obesity, insulin resistance, hyperlipidaemia and steatohepatitis compared to their wild-type littermates (Hirschey et al., 2011). The importance of SIRT3 in metabolic health has been also demonstrated in humans where a single nucleotide polymorphism in the SIRT3 gene has been associated with increased susceptibility to develop the metabolic syndrome (Hirschey et al., 2011). In support of the role of SIRT3 and mitochondrial protein acetylation in the regulation of energy metabolism, SIRT3 has been reported to regulate mitochondrial fatty acid β-oxidation *via* deacetylation of long-chain acyl-CoA dehydrogenase, which, when dysregulated, results in defective fatty acid oxidation (Hirschey et al., 2010). Moreover, SIRT3 appears to regulate energy metabolism and, particularly, fatty acid β-oxidation, by regulating AMPK phosphorylation and *PGC 1α* expression as demonstrated by the decrease in phosphorylated AMPK:total AMPK ratio and the downregulation of *PGC 1α* in the skeletal muscle of SIRT3 KO mice (Palacios et al., 2009). Furthermore, SIRT3 KO mice exhibited increased insulin resistance, which is underpinned by defective insulin-induced glucose uptake in skeletal muscle (Lantier et al., 2015). Thus, considering the association between defective fatty acids β-oxidation, lipotoxicity and insulin resistance, targeting SIRT3 and mitochondrial protein acetylation may represent a promising approach in enhancing insulin sensitivity and improving metabolic health.

## Mitochondrial Dynamics

Mitochondrial function is further regulated by acute changes in mitochondrial architecture, termed mitochondria dynamics, which encompasses cycles of fusion and fission and is crucial to allow the cells to respond to short-term metabolic perturbations (Wai and Langer, 2016). Despite mitochondria being classically portrayed as single isolated organelles, they are highly dynamic and capable of engaging in intimate interactions with other cellular components and dividing (fission) or fuse joining (fusion) depending on the metabolic status of the cell (McInnes, 2013). The maintenance of a healthy cellular mitochondrial network is dependent on the balance between fusion and fission cycles (Westermann, 2010), which in turn are governed by cellular metabolic perturbations and metabolic demand (Wai and Langer, 2016) and respond to fluctuation in the energy status of the cell (Liesa and Shirihai, 2013) depending on the cell type. Particularly, absolute or relative energy deficits, underlain by a drop in ATP levels or an increase in ATP demand, trigger mitochondrial fusion (Liesa and Shirihai, 2013; Hesselink et al., 2016), which consists in the fusion of the outer and inner mitochondrial membranes orchestrated by mitofusin-1 and mitofusin-2 and optic atrophy protein 1, respectively (Figure 3; Cipolat et al., 2004). On the contrary, an excess in substrates that fuel oxidative metabolism induces a shift towards mitochondria fission, which is associated with an increase in ROS production, impaired oxidative phosphorylation and depletion of mtDNA (Liesa and Shirihai, 2013). Mitochondria fission is regulated by dynamin-1-like protein and its cognate outer membrane receptor: mitochondrial fission 1 protein (Wai and Langer, 2016). Mitochondrial dynamics is intimately linked to mitochondrial function. Particularly, mitochondrial fusion is positively associated with increased energy efficiency and ATP production, while a shift towards mitochondrial fission results in a drop in mitochondrial efficiency and a concomitant increase in ROS production (Liesa and Shirihai, 2013; Wai and Langer, 2016; Lin et al., 2018). Mitochondrial dynamics is crucial to maintain healthy mitochondria and control their quality. Indeed, mitochondria fusion allows the exchange of damaged mtDNA with intact mtDNA resulting from the intimate interaction between isolated mitochondria. On the contrary, mitochondria fission promotes the removal of dysfunctional mitochondria *via* an autophagic process termed mitophagy (Westermann, 2010). Thus, considering the importance of mitochondrial dynamics in maintaining and promoting mitochondria health and allowing the cells to adapt and respond to metabolic challenges, it appears evident that disruption of mitochondrial dynamics compromises mitochondrial quality and cellular bioenergetics. In support of this paradigm, dysregulation of mitochondria dynamic with a shift towards fission promotes metabolic dysfunction as demonstrated by the onset of obesity and insulin resistance following the ablation of fusion protein in mice (Quiros et al., 2012; Sebastian et al., 2012). Furthermore, increased mitochondrial fission and consequent mitochondrial fragmentation have been associated with increased ROS production, mitochondrial depolarisation, impaired ATP production and decreased insulin-dependent glucose uptake in C2C12 murine cell line (Jheng et al., 2012) as well as increased mitochondrial ROS and impaired insulin signalling in cybrids (Lin et al., 2018), which are further evidence for the deleterious effect of unbalanced mitochondrial dynamics on metabolic health. *Mitofusin-2*, a key driver of mitochondria fusion, was downregulated in humans with obesity or T2DM, suggesting a decrease in mitochondrial fusion in these individuals (Bach et al., 2005). Nonetheless, *mitofusin-2* transcript levels increased upon weight loss (Bach et al., 2005), suggesting defects in mitochondrial dynamics may be driven by nutrient and energy oversupply and, most importantly, that these abnormalities are reversible. An imbalance in mitochondrial dynamics with a shift towards fission also negatively impacts on fatty acid β-oxidation, which in turn has been described as a pivotal metabolic defect in obesity and insulin resistance (Kelley et al., 1999; Simoneau et al., 1999) and contributes to the accumulation of lipotoxic lipid species and insulin resistance. In support of this, a fusion-shifted dynamics has been associated with an increase in fatty acid utilisation (Lionetti et al., 2014) putatively preventing lipotoxicity.

Mitochondria, as described so far, are able to adapt to changes in energy demand and metabolic substrates availability by undergoing cycles of fusion and fission. However, mitochondrial dynamics also includes mitochondria-organelle interactions including plasma membrane, lysosome and the endoplasmic reticulum (ER). Particularly, the dysregulation of the interactions between mitochondria and the ER has been reported to be implicated in the pathogenesis of insulin resistance (Flamment et al., 2012; Sebastian et al., 2012; Tubbs et al., 2018). Mitochondria-ER contact points, referred to as mitochondria-associated ER membranes (MAMs), are the sites where Ca^2+^, lipid and metabolite exchange occur, thus representing pivotal points of interaction for the regulation of oxidative metabolism (Jouaville et al., 1999). The interaction between mitochondria and ER has been demonstrated in mouse and human primary hepatocytes and in mice (Tubbs et al., 2014; Theurey et al., 2016). The disruption of the MAMs in the liver promotes insulin resistance suggesting MAM integrity being required for insulin signalling (Tubbs et al., 2014). However, this remains highly controversial with energy and nutrient overload leading to increased formation of MAMs, which in turn, by driving Ca^2+^ accumulation in the mitochondria, results in dysregulation of mitochondrial oxidative metabolism, increased ROS production and impeded insulin signalling (Arruda et al., 2014). Remarkably, the interaction between mitochondria and the sarcoplasmic reticulum (SR) has been confirmed in skeletal muscle (Tubbs et al., 2018; Thoudam et al., 2019). Not surprisingly, also in consideration of the pivotal role of skeletal muscle in the regulation of glucose homeostasis, the dysregulation of MAMs may contribute to skeletal muscle insulin resistance in mice and humans (Tubbs et al., 2018; Thoudam et al., 2019). Mitochondria-SR interactions have been reported to be reduced in the skeletal muscle of high-fat or genetically induced obese and diabetic mice as well as in human primary myotubes treated with palmitic acid (Tubbs et al., 2018), thus supporting the relationship between a reduction in MAMs and insulin resistance. Moreover, overexpression of FATE1, an organelle uncoupler, reduces mitochondria-SR interactions and dampens insulin signalling both *in vivo* and *in vitro*, whereas inducing organelle coupling protects human myotubes from palmitic acid-induced insulin resistance (Tubbs et al., 2018). Importantly, mitochondria-SR miscommunication develops before the onset of mitochondrial dysfunction and represents an early event in diet-induced insulin resistance (Tubbs et al., 2018) indicating that the MAM reduction may represent a link between mitochondrial dysfunction and insulin resistance in skeletal muscle. Nonetheless, the role of mitochondria-SR interactions in promoting insulin signalling in skeletal muscle is emerging as a matter of contention, especially in consideration of a recent report supporting the possibility, that instead of a decrease, an increase in MAM formation dampens insulin signalling in skeletal muscle. This relies on mitochondrial Ca^2+^ accumulation, which is underpinned by the activation of pyruvate dehydrogenase kinase 4 (PDK4) and the subsequent stabilisation of key proteins involved in mitochondria-SR interactions, namely inositol 1,4,5-triphosphate receptor 1, 75-kDa glucose-regulated protein and voltage-dependant anion channel 1 (Thoudam et al., 2019). In support of this, pharmacological inhibition or deletion of the gene encoding for PDK4 led to a decrease in MAM formation, which resulted in an increase in insulin signalling in both mice and C2C12 myotubes (Thoudam et al., 2019). The downstream event being held responsible for the deleterious metabolic effect of augmented mitochondria-SR interactions is the accumulation of Ca^2+^ in the mitochondria (Thoudam et al., 2019). Ca^2+^ plays an important role in promoting fatty acid catabolism by stimulating downstream enzymes in the Krebs cycle indicating that the formation of MAMs and the subsequent increase in mitochondrial Ca^2+^ may exert a short-term beneficial effect by increasing fatty acid β-oxidation. Nonetheless, continuous fatty acid overload may lead to uncontrolled and sustained formation of MAMs, which, by increasing mitochondrial Ca^2+^, leads to increased ROS production and mitochondrial dysfunction (Thoudam et al., 2019). The discrepancies between the aforementioned studies may arise from the use of two diverse experimental diets with Tubbs et al. using a high-fat, high-fructose diet [36% fat, 35% carbohydrate (50% sucrose) and 19.8% protein] (Tubbs et al., 2018) while Thoudam and co-workers using a high-fat diet [60% fat, 20% carbohydrates (58% sucrose) and 20% protein, D12492, Inc., New Brunswick, USA] (Thoudam et al., 2019). Nonetheless, independently on this contention, MAM formation appears to act as an important regulator of mitochondrial function and insulin sensitivity, further highlighting the pivotal role of mitochondrial dynamic in the regulation of mitochondrial function and insulin signalling.

## Mitochondria Function and Supercomplex Formation

Mitochondrial function is also regulated by the formation of mitochondrial supercomplexes. The mitochondrial electron transport chain is a composite multiprotein system embedded in the mitochondrial inner membrane and is responsible for harvesting energy from metabolic fuels and transforming it into ATP. It encompasses four complexes (complexes I–IV), which transport the electrons derived from the oxidation of NADH and FADH_2_, which in turn are generated during glycolysis, beta-oxidation and the Krebs cycle. The final acceptor of the electrons transported *via* the electron transport chain is O_2_, which is converted to water. Coupled with the transport of electron is the pumping of protons from the inner mitochondrial membrane resulting in the generation of an electro-chemical gradient, which is finally used by the ATP synthase (complex V) to generate ATP. The electron transport chain complexes despite existing in single free moving proteins, due to their high plasticity, are able to assemble into multimeric super assembled structures termed supercomplexes (Lapuente-Brun et al., 2013; Kühlbrandt, 2015). The most abundant mitochondrial supercomplex is made up of complexes I, III and IV (molar ratio: I + III2 + IV1 − 4), which is generally referred to as respirasome (Figure 3; Lapuente-Brun et al., 2013). Respirasomes allow the cell to autonomously respire in the absence of mobile electron carriers such as ubiquinone and cytochrome C and decrease the diffusion distance between complexes, thereby improving the efficiency of electron transport and lowering ROS generation (Genova and Lenaz, 2014; Cogliati et al., 2016; Moreno-Loshuertos and Enríquez, 2016). Remarkably, mitochondrial electron transport chain supercomplexes containing complex I, II, and IV were reported to be decreased in individuals with T2DM relative to non-diabetic controls (Figure 3), which was associated with a decrease in maximal ADP-stimulated respiration (Antoun et al., 2015). Most importantly, a decrease in mitochondrial respiration supported by complex I and complex II substrates or octanoyl carnitine significantly correlated with glycated haemoglobin (Antoun et al., 2015). Instead, mitochondria electron transport chain supercomplex assembly is promoted by exercise (Figure 3) and resulted in increased maximal O_2_ consumption and mitochondrial respiratory capacity as well as increased reliance on fatty acid oxidation during exercise (Greggio et al., 2017). Thus, defective mitochondrial supercomplex assembly represents another putative mechanism linking impaired mitochondrial oxidative metabolism, insulin resistance and lipotoxicity and provides a novel target to mimic the beneficial effects of exercise on metabolic health.

## Dietary Long-Chain Saturated Fatty Acids as Key Drivers of Mitochondrial Dysfunction

Long-chain saturated fatty acids have been widely described as detrimental to metabolic health by promoting both metabolic inflammation and lipotoxicity (Summers, 2006; Sears and Perry, 2015; Sergi et al., 2018a,b). As discussed in the previous sections, lipid overconsumption appears to be detrimental to mitochondrial health. In support of this, the overconsumption of fat (50% of total energy intake) for 3 days was sufficient to affect the expression of genes involved in mitochondria electron transport chain and mitochondrial biogenesis in skeletal muscle including members of mitochondrial complex I and II as well as PGC 1α and β (Sparks et al., 2005). The negative impact of fat oversupply to skeletal muscle has been confirmed by acute elevation of circulating fatty acid levels, which also decreased the expression of PGC 1α and β alongside other genes involved in mitochondrial metabolism (Richardson et al., 2005; Hoeks et al., 2006) further supporting the harmful effects of increased fatty acid availability on mitochondria health. This evidence was confirmed in Wistar rats in which a high-fat diet induced a decrease in mitochondrial respiration and ATP production in the soleus muscle, an effect which was also induced by a high-fructose diet (Chanseaume et al., 2006). Furthermore, feeding mice a high-fat diet for 8 or 16 weeks has been reported to promote impaired fasting glucose and impaired glucose tolerance, which was parallel with a decrease in mitochondria number, ATP synthesis and mitochondrial membrane potential in skeletal muscle (Xu et al., 2019). A high-fat diet also increases muscle lipids and acylcarnitines, which correlated with insulin resistance and defective *in vivo* muscle mitochondrial oxidative metabolism (Wessels et al., 2015). Thus, it appears that increased lipid supply to skeletal muscle represents a key driver of mitochondrial dysfunction (Schrauwen et al., 2010). Particularly, lipotoxicity and ROS have been proposed as the mediators of fatty acid oversupply-induced mitochondrial dysfunction (Schrauwen et al., 2010). When mitochondrial oxidative capacity fails to match increased fatty acid supply to skeletal muscle, fatty acids accumulate in the proximity of mitochondria and can then translocate to the mitochondrial matrix *via* a flip-flop mechanism, which bypass both acyl-CoA synthase and carnitine palmitoyl acyl transferase 1 resulting in the accumulation of non-metabolisable fatty acids in the mitochondria (Ho et al., 2002; Schrauwen et al., 2010), a phenomenon which has been associated with insulin resistance (Koves et al., 2008). These fatty acids may undergo oxidative damage by ROS with the consequent formation of lipid peroxides, which in turn contribute to oxidative damage to mtDNA and proteins. In support of this possibility, despite obese insulin resistant subject presenting similar amount of intracellular triglycerides as endurance athletes, the degree of lipid peroxidation was 4.2-fold higher in obese insulin resistant individuals than endurance athletes (Russell et al., 2003) supporting the role in lipid peroxides not only in promoting mitochondrial dysfunction but also as a putative link between fatty acid over-supply, mitochondrial dysfunction and insulin resistance. Besides lipid peroxides, ceramide also takes the blame for the detrimental effect of fatty acid overload on mitochondrial function. Indeed, ceramide apart from its deleterious role in insulin signalling pathway (Summers, 2006; Chavez and Summers, 2012) also negatively impacts upon mitochondrial function. Particularly, treatment of muscle cell with the cell permeable ceramide C2 induced mitochondrial fission underpinned by the upregulation of dynamin-related protein 1. Furthermore, ceramide treatment resulted in a decrease in mitochondrial oxygen consumption paralleled by an increase in H_2_O_2_ as well as an impairment of insulin signalling in myotubes as demonstrated by a decrease in AKT phosphorylation. Remarkably, the detrimental effect of ceramide on mitochondrial bioenergetics and dynamics as well as insulin signalling were prevented by inhibiting mitochondrial fission (Smith et al., 2013). Hence, a balanced mitochondrial dynamics is pivotal in preserving insulin sensitivity and preventing the deleterious effect of lipid overload on mitochondrial bioenergetics in skeletal muscle and possibly other tissues such as the hypothalamus and the liver. Finally, a high-fat diet has also been reported to downregulate the protein levels of SIRT3 (Palacios et al., 2009), which represents a key regulator of mitochondrial energy metabolism (Palacios et al., 2009; Hirschey et al., 2010, 2011; Anderson and Hirschey, 2012). Thus, the deleterious effect on a high-fat diet and the consequent increase in lipid supply to skeletal muscle is not only limited to the dysregulation of the expression of proteins and genes directly involved in mitochondrial function, dynamics and biogenesis, but also affects the post-translational modifications of these proteins by modulating upstream regulators of mitochondrial function as in the case of SIRT3. Nonetheless, considering indistinguishably all fatty acids as detrimental is rather simplistic, especially considering that the impact of fatty acids on metabolic health is dictated by their chemical characteristics with unsaturated fatty acids, medium- and short-chain fatty acids being proven as beneficial to metabolic health, compared to long-chain saturated fatty acids (Roche, 2005; Holland et al., 2007; van Dijk et al., 2009; Olefsky, 2012; Canfora et al., 2015; McCarty and DiNicolantonio, 2016). Mitochondria are not immune from the deleterious effect of long-chain saturated fatty acids. Indeed, the exposure to long-chain saturated fatty acids is not without consequences on mitochondrial health as demonstrated in the L6 rat cell line in which palmitic acid, the main long-chain saturated fatty acid in the Western diet, induced mitochondrial dysfunction associated with increased mtDNA damage, induction of c-Jun N-terminal kinases (JNK), apoptosis, and insulin resistance all underlain by increased mitochondrial ROS generation (Yuzefovych et al., 2010). Furthermore, palmitic acid also affected mitochondrial biogenesis by decreasing PGC 1α protein levels and the expression of TFAM in L6 skeletal muscle cells (Yuzefovych et al., 2010). The effect of long-chain saturated fatty acids was confirmed in C2C12 muscle cell line in which both palmitic and stearic acid challenge induced mitochondrial dysfunction characterised by mitochondria membrane hyperpolarisation and defective ATP generation (Hirabara et al., 2010). Mitochondrial dynamics is also susceptible to long-chain saturated fatty acids thereby representing a further target which bridges the gap between increased long-chain saturated fatty acid availability and mitochondrial dysfunction. In support of this notion, exposure of C2C12 muscle cells to palmitic acid resulted in increased mitochondria fission as demonstrated by increased mitochondrial fragmentation orchestrated by the upregulation of dynamin-related protein 1 and mitochondrial fission 1 protein (Jheng et al., 2012). Not surprisingly, this process was associated with increased oxidative stress and loss of ATP synthesis (Jheng et al., 2012) as previously described for ceramide (Smith et al., 2013) indicating that this lipotoxic metabolite may represent the downstream effector of the detrimental effect of long-chain saturated fatty acid on mitochondrial health. Remarkably, exposure of muscle cells to long-chain saturated fatty acids, apart from inducing mitochondrial dysfunction, also impairs insulin sensitivity, further strengthening the relationship between mitochondrial function and insulin-induced glucose metabolism.

## Omega-3 Polyunsaturated Fatty Acids (PUFAs) and Mitochondrial Function

Diverse dietary fat sources may differently affect mitochondrial function and insulin resistance development in skeletal muscle *via* different mechanisms (Putti et al., 2015a,b). As described earlier, long-chain saturated fatty acids play a key role in promoting insulin resistance by impairing mitochondrial bioenergetics and dynamic behaviour. On the contrary, omega-3 PUFAs have been reported to improve skeletal muscle insulin sensitivity by modulating mitochondrial function. Omega-3 PUFAs are extremely flexible molecules due to the long tail of double bonds typical of their carbon chain and include the essential α-linoleic acid (ALA) and longer-chain fatty acids, eicosapentaenoic (EPA) and docosahexaenoic (DHA). In recent years, studies in rodents and humans have indicated that omega-3 PUFAs elicit beneficial effects on metabolic health by reducing obesity and improving insulin resistance with a mechanism, which relies, at least in part, on their ability to increase fat oxidation and energy expenditure and reduce fat deposition (Xu, 2015; Lalia and Lanza, 2016). Current knowledge on the potential role of omega-3 fatty acids to improve insulin sensitivity has been recently reviewed by Lalia and Lanza (2016). Moreover, an up-to-date commentary on the potential mechanisms by which omega-3 PUFAs may exert beneficial effects on insulin sensitivity at the cellular level has been the focus on a recent review describing the link among mitochondria, ER stress and inflammatory pathways (Lepretti et al., 2018).

Omega-3 PUFAs have been shown to increase mitochondrial content in skeletal muscle as demonstrated by the upregulation of transcription factors, which govern mitochondrial biogenesis, including PGC 1α and NRF1, in the skeletal muscle of mice fed a high-fat diet (60% energy from fat) supplemented with fish oil (3.4% kcal from n-3 PUFAs) for 10 weeks (Lanza et al., 2013). In addition, several studies suggested that omega-3 PUFAs can prevent or reverse the impairments in skeletal muscle mitochondrial function by increasing fatty acid oxidation. An increase in mitochondrial carnitine palmitoyl transferase 1 (CPT-1, the rate limiting enzyme in fatty acid β-oxidation) activity has been shown in skeletal muscle of rats fed a high-fat diet (200 g fat/kg) containing menhaden (fish) oil (Power and Newsholme, 1997). This was further confirmed in rats fed a high-fat diet supplemented with 10% v/w omega-3 PUFAs from fish oil for 6 weeks, which induced an upregulation of mitochondrial CPT-1 and induced fatty acid β-oxidation, with both being dependent upon the activation of AMPK in skeletal muscle (Motawi et al., 2009). The increased fatty acid utilisation is likely to contribute to a decrease in ectopic lipid accumulation playing an important role in counteracting lipotoxicity and insulin resistance onset. Noteworthy, it has also been reported an increased expression of uncoupling protein 3 (UCP3) in skeletal muscle of male Fisher 344 rats fed a diet containing 40% of energy in the form of fish oil for 6 weeks (Baillie et al., 1999). Mitochondrial uncoupling results in a decrease in mitochondrial energy production efficiency leading to an increase in fatty acid catabolism and lower ectopic lipid accumulation, which may represent a further mechanism by which omega-3 PUFAs exert their beneficial effects on insulin sensitivity. Moreover, the upregulation of UCP3 may be beneficial in counteracting ROS production and the subsequent impairment of insulin signalling pathways, with a similar mechanism being suggested for omega-3 PUFA effects on liver mitochondrial function and prevention of high-fat diet-induced insulin resistance (Lionetti et al., 2014).

As previously discussed, the role of mitochondrial morphology and dynamic behaviour in determining mitochondrial dysfunction and insulin resistance onset has been the object of growing interest in the recent years. Therefore, another aspect to be considered when analysing the beneficial effect of omega-3 PUFAs is their impact on mitochondrial dynamic behaviour and morphology. DHA (25 μM for 4 h) induced a higher proportion of large and elongated mitochondria in cultured L6 myocytes associated with a downregulation of genes involved in promoting mitochondrial fission: DRP1 and Fis1 (Casanova et al., 2014). Remarkably, the replacement of lard with fish oil (40% fat J/J) in chronic (6 weeks) high-fat feeding positively affected mitochondrial dynamic behaviour in association with the improvement of insulin resistance in both skeletal muscle and liver in Wistar rats (Lionetti et al., 2013, 2014). In skeletal muscle, a higher immunoreactivity for Mfn2 and OPA1 proteins was observed in high-fish oil fed rats compared to high-lard fed littermates suggesting a shift in mitochondrial dynamics towards fusion, which was further confirmed by the weaker immunostaining for DRP1 and Fis1 and a prominent presence of fusion events observed by electron microscopy in high-fish oil relative to high lard fed rats (Lionetti et al., 2013). This effect of fish oil on mitochondrial dynamic behaviour was associated with an improvement in skeletal muscle insulin signalling as demonstrated by normalisation of IRS1 and pIRS (Tyr632) immunoreactivity in skeletal muscle to the levels of the control diet and improved systemic insulin sensitivity (Lionetti et al., 2013).

Noteworthy, the tendency to mitochondrial fusion induced by dietary omega-3 PUFAs, compared to dietary long-chain saturated fatty acids, may also be related to the well-known anti-inflammatory effect of omega-3 PUFAs as opposed to long-chain saturated fatty acid, which, instead, has been reported to be pro-inflammatory (Sergi et al., 2018b). In support of this, a link between mitochondrial dynamics and inflammation exists as shown by the downregulation of *Mfn2* gene expression induced by TNF-α in cells in culture (Bach et al., 2005). Thus, it can be speculated that the pro-inflammatory effects of long-chain saturated fatty acids may be responsible for the reduction in *Mfn2*, which in turn may contribute to the development of insulin resistance as discussed previously. On the contrary, the anti-inflammatory effect of omega-3 PUFA may contribute to induce *Mfn2* expression, thereby counteracting insulin resistance. In agreement with the pro-fusion effect of omega-3 PUFAs on skeletal muscle mitochondria, the positive effect of omega-3 PUFAs on inflammation and insulin resistance was also associated with improved mitochondrial function and a shift towards fusion processes in rat liver (Lionetti et al., 2014), suggesting the protective effect of omega-3 PUFAs against insulin resistance relying on overlapping mechanisms in diverse peripheral tissues.

## Caloric Restriction, Intermittent Fasting, and Mitochondrial Function

Caloric restriction (CR) improves insulin sensitivity and delays the onset of metabolic and age-related diseases in a wide variety of organisms, including non-human primates. One theory through which CR is theorised to improve health and longevity is by a reduced “rate of living” and oxidative damage. However, the impacts of CR on mitochondrial function and bioenergetics are controversial.

A number of studies have shown that CR increases mitochondrial biogenesis (Nisoli et al., 2005; Lopez-Lluch et al., 2006) and mitochondrial efficiency (Nisoli et al., 2005) and reduces mitochondrial production of ROS (Bevilacqua et al., 2005). However, this is not consistent across studies or across tissues (Hancock et al., 2011; Lanza et al., 2012; Walsh et al., 2014). Hancock et al. could not detect any changes in mRNA or protein levels of markers of mitochondrial biogenesis, or citrate synthase activity in muscle from rats that underwent 30% CR for 14 weeks (Hancock et al., 2011). Similarly, long-term CR did not alter any markers of mitochondrial biogenesis, although CR prevented age-related loss of mitochondrial oxidative capacity and efficiency in isolated mitochondria and in muscle fibres, and reduced oxidative damage (Hancock et al., 2011). In a systematic review of over 157 studies on the effects of CR on mitochondrial ROS production, Walsh et al. reported that 46% of studies report a reduction in ROS production in muscle, and 60% of studies detected a reduction in mitochondrial ROS production in brain (Walsh et al., 2014). Of note, significant reduction in ROS production was more likely if the duration of the caloric restriction exceeded 20 months.

Discrepancies in outcomes between studies could be due to differences in the tissue examined, the duration of CR, the degree of energy restriction, the dietary fat load or source imposed. The mouse strain under investigation, and gender, is also likely to affect outcomes. Mulvey et al. (2017) selected female mouse strains who either have increased, no change, or reduced lifespan in response to 40% CR. CR for 10 months did not alter the nDNA:MtDNA ratio, protein levels of markers of mitochondrial biogenesis in liver or skeletal muscle, in any of the mouse strains that were investigated. Reduced oxygen consumption rates in isolated mitochondria from hepatocytes were observed in animals whose lifespans are shortened by CR. This study could not demonstrate a beneficial effect of CR on mitochondrial dysfunction in the long-lived strain. The effects on mitochondrial ultrastructure and markers of mitochondrial fission and fusion have been investigated in recent studies of CR. Khraiwesh et al. (2013) showed that 6 months of CR increased hepatocyte cristae number and resulted in a more spherical mitochondrial shape. They also observed that proteins related to mitochondrial fission (e.g., Fis1 and Drp1) increased with CR, but there was no change in markers of mitochondrial fusion (Mfn1, Mfn2, and OPA1).

In humans, the impacts of caloric restriction on mitochondrial function are also controversial. Civitarese et al. (2007) reported that markers of mitochondrial biogenesis and mtDNA content were stimulated in response to 6 months of 25% CR in 12 men and women who were overweight. However, there was no change in key enzymes involved in mitochondrial activity. In another study, caloric restriction for 16 weeks reduced body weight and improved insulin sensitivity but did not alter mitochondrial volume, NADH oxidase or β-hydroxyacyl CoA dehydrogenase (β-oxidation pathway) in seven obese individuals. However, citrate synthase, a marker of mitochondrial content was increased (Menshikova et al., 2017). Similarly, 16 weeks of CR increased insulin sensitivity in 11 individuals who were obese but did not affect skeletal muscle mitochondrial oxidative capacity or oxidant emissions (Johnson et al., 2016). These studies were conducted in small cohorts. Recently, Sparks et al. (2016) investigated the effects of 12 months of 25% caloric restriction vs. control on mitochondrial function in 51 individuals who were overweight, but not obese. In this study, CR reduced intramyocellular lipid in soleus, and mRNA levels of genes involved in lipogenesis and lipid transport. However, there was no change in mitochondrial capacity as measured by ATPmax, or in coupling efficiency as measured by the P/O ratio, or in mRNA levels of markers of mitochondrial biogenesis. Contrary to expectations, individuals who had a more coupled phenotype at baseline were able to better improve mitochondrial function in response to CR.

Intermittent fasting (IF) is a dietary alternative to CR that is characterised by intermittent periods of fasting (typically 24 h), interspersed with ad-libitum (AL) access to food. Similar to CR, IF improves multiple markers of health and increases longevity (Fontana and Partridge, 2015). In contrast to CR, body weight is not changed, or modestly reduced, in chow fed IF vs. AL animals (Goodrick et al., 1990). The impacts of intermittent fasting on mitochondrial function have only been investigated by a handful of studies. Chausse et al. (2015) observed no change in markers of mitochondrial biogenesis, or in the oxidative capacity in isolated mitochondria from brain, or heart in rats who underwent every other day feeding for 4 weeks. Increases in markers of oxidative damage were observed in liver and brain, but IF provided protection against oxidative damage in the heart. No difference in mitochondrial bioenergetics or redox homeostasis was observed in skeletal muscle. By contrast, Singh et al. (2012) have reported protection against oxidative damage in the brain by short term, late onset, IF. A separate study reported IF enhanced mitochondrial respiration in white adipose tissue, but did not impact liver, skeletal muscle or brown adipose tissue (Boutant et al., 2016). The effects of IF on mitochondrial ultrastructure and dynamics are unclear.

## The Effect of Amino Acids and High-Protein Diet on Mitochondrial Function

Numerous studies have fed animal and humans varying types of protein-rich supplements and demonstrated modest associations between increased postprandial plasma amino acid concentrations, particularly the branched chain AAs (i.e., leucine, isoleucine and valine), with physiological outcomes including an increased muscle protein synthesis, release of some gut hormones (particularly, GLP-1 and GIP), insulin and a reduced energy intake (Floyd et al., 1966; Nilsson et al., 2007; Veldhorst et al., 2009). More recently, essential and/or branched chain amino acids have been shown to support cardiac and skeletal muscle through increased mitochondrial biogenesis and function in both mice and humans (D’Antona et al., 2010; Tatpati et al., 2010; Valerio et al., 2011).

Given impaired mitochondrial function in skeletal muscle is one of the major predisposing factors to metabolic diseases such as insulin resistance, T2DM and cardiovascular diseases, understanding the effects of specific amino acids on mitochondrial function is pivotal to determining what type of dietary proteins are optimal for preventing disease associated with mitochondrial dysfunction. While our understanding regarding how dietary protein or specific amino acid supplements promote mitochondrial biogenesis in metabolically active tissues and the exact molecular mechanisms through which they occur remains limited, it has been postulated that amino acid supplementation induces mitochondrial biogenesis to promote catabolism of amino acids themselves (Valerio et al., 2011). This is plausible because amino acids are used as precursors of tricarboxylic acid (TCA) cycle intermediates and produce approximately 10–15% of total metabolic energy in animals apart from serving as a metabolic fuel during exercise. In support for a putative effect of amino acids on mitochondria function, a high-protein diet has been shown to promote a higher rate of fatty acid oxidation than a high-carbohydrate counterpart (Raben et al., 2003) and to increase postprandial fat oxidation following a high-protein meal (Labayen et al., 2004), suggesting that protein and most likely branched chain amino acids may boost oxidative metabolism. Although the effect of amino acids on mitochondrial function is under-represented in the literature, there is emerging evidence that leucine can increase cell respiration, increase mitochondria biogenesis and upregulate *PGC 1α* and *SIRT1* (Sun and Zemel, 2009). This was confirmed in animal models with branched chain amino acid supplementation promoting mitochondrial biogenesis and inducing the expression of SIRT1 in skeletal muscle (D’Antona et al., 2010), which was also associated with an increase in skeletal muscle fatty acid catabolism *in vitro* (Liang et al., 2014). Nonetheless, despite the evidence from *in vitro* and animal models, the effect of amino acid supplementation and high-protein diets on human skeletal muscle mitochondrial function remains elusive. Thus, future research is required to better understand the synergistic actions of all amino acids on muscle mitochondrial function and insulin resistance, as well as shed the light on the molecular mechanisms through which they exert their effects.

## Food Bioactive Derivatives and Mitochondrial (DYS)Function

Bioactive compounds are commonly referred to as a non-nutritive compounds that are present in very small quantities in foods but do have tremendous potential to produce significant improvements in human health (Naumovski, 2014; Christenson et al., 2016). Furthermore, the use of food bioactive derivatives, predominately from plant-based products, has long been described as particularly favourable as it provides a relatively easy and affordable method to incorporate nutraceuticals in the diet. The health promoting effect of these bioactive molecules also extends to mitochondria and may represent a valuable nutritional tool to prevent or mitigate the metabolic aberrations underpinning mitochondrial dysfunction. In this regard, the bioactives, which have been most widely described for their effect on mitochondria (dys)function, include, but are not limited to, Coenzyme Q10, resveratrol and quercetin.

### The Coenzyme Q10

The Coenzyme Q10 (CoQ10) is a key component of the electron-transport chain (ETC) and is commonly referred to as ubiquinone. Contrarily to the other bioactives described in this section, CoQ10 is particularly abundant in animal food products. Besides its role as an electron transporter from complex I and II to complex III, CoQ10 is also a potent antioxidant, which protects cells from oxidative damage. Thus, ubiquinone supplementation can positively modulate mitochondria function by supporting electron transport in the ETC on one hand and prevent mitochondrial oxidative damage on the other (Shen and Pierce, 2015). Furthermore, there is a close relationship between mitochondria dynamics and CoQ10, with MTF2 being required for the synthesis of CoQ10 and ubiquinone itself being able to rescue the reduction in respiratory function resulting from MTF2 deficiency (Mourier et al., 2015). Of note, CoQ10 deficiency has been observed in individuals with T2DM, and considering the role of mitochondrial dysfunction in the pathogenesis of insulin resistance and oxidative stress, it can be speculated that CoQ10 supplementation may improve glycaemic control *via* a direct effect on mitochondrial function (Shen and Pierce, 2015).

### Quercetin

Quercetin is a polyphenol, which belongs to the class of flavonoids and is particularly abundant in apples, onions, peppers, berries and leafy green vegetables. Quercetin has widely been reported for its favourable effect on skeletal muscle and mitochondria biogenesis as well as function *via* the activation of the SIRT1-AMPK-PGC 1α axis (Davis et al., 2009). Indeed, this polyphenol has been reported to activate the AMPK and SIRT1 (Howitz et al., 2003; Hawley et al., 2010) which, as described above, are pivotal regulators of mitochondrial oxidative metabolism. Furthermore, quercetin can stimulate mitochondria oxidative metabolism by directly decreasing ATP:AMP ratio, which in turn results in the activation of AMPK and its downstream catabolic pathways (Dorta et al., 2005). Quercetin has been reported to modulate fatty acid metabolism *in vitro* with an increase in fatty acid β-oxidation being reported in C2C12 myotubes, hepatocytes and Hela cells (Suchankova et al., 2009; Eid et al., 2010). Nonetheless, these effects on lipid metabolism were not confirmed *in vivo* as demonstrated by quercetin supplementation failing to affect fatty acid β-oxidation or respiratory exchange ratio (Dumke et al., 2009). Finally, despite quercetin being able to modulate pivotal pathways involved in mitochondria biogenesis and oxidative metabolism in rodents (Davis et al., 2009), its effect on mitochondria function and fatty acid catabolism in human skeletal muscle remains to be fully elucidated.

### Resveratrol

Resveratrol (trans-3,4′,5-trihydroxystillbene) is a stilbenoid polyphenol, which has been found predominately in grapes, nuts and berries. Despite the initial interest towards this bioactive molecules primarily focused on its putative role in increasing longevity, resveratrol has emerged in the recent years for its beneficial effects on metabolic health due to its ability to modulate mitochondria function and biogenesis and oxidative metabolism (Christenson et al., 2016). With regard to its effects on mitochondria biology, resveratrol has been reported to promote mitochondria biogenesis by activating SIRT1, which in turn, by deacetylating PGC 1α, induces its transcriptional activity resulting in an increase in mitochondria number in mice (Lagouge et al., 2006). Furthermore, resveratrol has been shown to counteract the deleterious effect of a high-fat diet on metabolic and mitochondria health in rats. While a high-fat diet induced insulin resistance, downregulated SIRT1 and SIRT3, inhibited mitochondria biogenesis and decreased mtDNA, resveratrol countered high-fat diet-induced metabolic deterioration with a mechanisms which relayed albeit in part, on its beneficial effects on mitochondrial health and function (Haohao et al., 2015). Remarkably, the effect of resveratrol was confirmed in the first-degree relatives of type 2 diabetic individuals with resveratrol increasing mitochondrial respiration on octanoyl-carnitine (de Ligt et al., 2018). However, despite producing an *ex vivo* increase in mitochondrial respiration that may be dependent of its ability to increase AMPK activity (Kulkarni and Cantó, 2015), resveratrol did not induce an increase in insulin sensitivity in these individuals, suggesting that a small increase in mitochondrial function may not be sufficient to enhance insulin sensitivity if not supported by higher levels of physical activity (de Ligt et al., 2018).

Besides the aforementioned compounds, other food-derived bioactives have been described as potential regulator of mitochondrial function. The administration of a epicatechin-rich cocoa in type 2 diabetic individuals resulted in an increase in mitochondria biogenesis in skeletal muscle as demonstrated by an increased activation of the SIRT1-PGC 1α axis and an increase in the abundance of mitochondrial complex I and V (Taub et al., 2012). Coumestrol, a bioactive found in legumes, was also described for its ability to increase mitochondria biogenesis in cultured muscle cells underlined by an upregulation of electron transport chain proteins and transcriptional regulator of mitochondria biogenesis: PGC 1α and NFR-1 (Seo et al., 2014). The bioactives described thus far only represent a portion of the food bioactive molecules being described as positive modulator of mitochondrial function and biogenesis, a more comprehensive list of food bioactive derivatives able to increase mitochondrial function has been reviewed elsewhere (Serrano et al., 2016).

Thus, food bioactive derivatives can, not only improve mitochondrial function by directly scavenging ROS and protecting mitochondria from oxidative damage, but also activate intracellular signalling pathways known to modulate mitochondria function and biogenesis including AMPK, SIRT1 and NRF-1, which renders these molecules an attractive nutritional tool in metabolic health. However, studies investigating the effect of food bioactive derivatives on mitochondrial biology remain limited, and further investigation is warranted to identify novel food-derived molecules, also used in combination, which may be able to improve mitochondrial function and ameliorate metabolic health.

## Conclusions

Mitochondrial dysfunction has been widely described as a metabolic defect associated with insulin resistance and T2DM. Mitochondrial function is regulated at different levels, which include mitochondrial biogenesis, post-translational modification of mitochondrial protein, mitochondrial dynamics and supercomplexes formation with all these processes appearing to be dysregulated in type 2 diabetic individuals. However, whether these mitochondrial defects represent a cause or a consequence of insulin resistance in skeletal muscle remains to be fully elucidated. Nonetheless, independently on whether mitochondrial dysfunction represents a primary defect in the pathogenesis of insulin resistance, increasing mitochondrial function represents a promising approach to enhance insulin sensitivity. Indeed, athletes in light of their higher mitochondrial oxidative capacity relative to sedentary individuals appear to be protected, albeit in part, from lipid induced insulin resistance, confirming that interventions aimed at increasing mitochondrial function (i.e., exercise) represent a valuable therapeutic tool to improve skeletal muscle insulin sensitivity. However, exercise is not the only lifestyle intervention able to positively modulate mitochondrial function. In this regard, dietary nutrients such as omega-3 fatty acids, food bioactive derivatives and caloric restriction are emerging as promising nutritional tools to boost mitochondrial function and prevent and/or ameliorate the metabolic dysfunctions associated with mitochondrial dysfunction. Despite these advances in understanding the role of nutrition in mitochondrial function, dietary patterns and combination of nutrients and food bioactives able to restore mitochondrial oxidative capacity are still to be identified as it remains to be elucidated as to whether their putative effect on mitochondrial function translates into improved metabolic health.

## Author Contributions

All authors participated in the conception, design, writing and editing of this review article. All authors read and approved the final version of the manuscript.

### Conflict of Interest Statement

The authors declare that the research was conducted in the absence of any commercial or financial relationships that could be construed as a potential conflict of interest.

## Footnotes

^1^http://www.who.int/mediacentre/factsheets/fs311/en/
